# LncRNA OIP5-AS1 Promotes the Proliferation of Hemangioma Vascular Endothelial Cells via Regulating miR-195-5p/NOB1 Axis

**DOI:** 10.3389/fphar.2019.00449

**Published:** 2019-04-24

**Authors:** Jiayu Zhang, Tiancheng Zhao, Leilei Tian, Yezhou Li

**Affiliations:** ^1^Department of Endoscopic Center, China-Japan Union Hospital of Jilin University, Changchun, China; ^2^Department of Gastrointestinal Colorectal and Anal Surgery, China-Japan Union Hospital of Jilin University, Changchun, China; ^3^Department of Operating Room, China-Japan Union Hospital of Jilin University, Changchun, China; ^4^Department of Vascular Surgery, China-Japan Union Hospital of Jilin University, Changchun, China

**Keywords:** human hemangioma endothelial cells, lncRNA, OIP5-AS1, miR-195-5p, proliferation, apoptosis

## Abstract

**Background:** Hemangioma, which is a benign vascular neoplasm, has been demonstrated to be the most common tumor of infancy, resulting from aberrant proliferation of endothelial cells and pericytes.

**Objective:** The aim of this study was to determine the role of lncRNA OIP5-AS1 on the proliferation and metastasis of human hemangioma endothelial cells (HemECs).

**Methods:** In this study, we examined the expression of OIP5-AS1 and miR-195-5p in hemangiomas patients. The effects of knockdown or ectopic expression of OIP5-AS1 on the viability, proliferation and apoptosis of HemECs were explored. Rescue assays were performed to explore the effects of OIP5-AS1/miR-195-5p/NOB1 axis on biological behaviors in the HemECs.

**Results:** It was found that OIP5-AS1 was overexpressed in both hemangioma tissues and HemECs cells. OIP5-AS1 knockdown suppressed the proliferation, migration, and invasion of HemECs cell, while overexpression of OIP5-AS1 had opposite effects LncRNA OIP5-AS1 acted as a ceRNA through binding with miR-195-5p to upregulate NOB1 in HemECs.

**Conclusion:** Taken together, lncRNA OIP5-AS1 acted as a ceRNA to drive proliferation, migration and invasion of HemECs through regulating miR-195-5p/NOB1 axis.

## Introduction

Infant hemangioma (IH) is a benign neoplasm commonly seen in childhood, which is more common in premature infants and infants with low birth weight (Smith et al., 2017). There are three distinct clinical histological stages in IH: the early proliferative stage, when undeveloped blood vessels are rapidly growing; plateau stage and final regressive stage, with scattered capillaries and large drainage vessels (Csoma et al., 2017). IH is characterized by spontaneous regression of blood vessels. IH can cause defects in some cases, which is also the main cause of pathogenesis of children with IH (Ji et al., 2014; Goelz and Poets, 2015; Khan et al., 2017). IH treatments include propranolol (Smithson et al., 2017), pulsed dye laser (Chinnadurai et al., 2016), nadolol (Villalba-Moreno et al., 2015), etc., the effects of which have been improved a lot in recent years. Nevertheless, the main clinical problem up to date is still the deficiency of reliable parameters to aid treatment against lesion in patients with IH. Therefore, identification of the genetic and epigenetic changes in IH lesions is of great value to clarify the molecular mechanism of IH occurrence.

Long non-coding RNAs (lncRNAs) are a group of transcripts without protein coding ability (Sun et al., 2018). lncRNAs form diversified non-coding RNAs (ncRNAs), which are longer than 200 nucleotides without an open reading frame of effective length (Chen Q. et al., 2018). It was confirmed in previous studies that lncRNAs are involved in almost all biological behaviors, including epigenetics, transcription and post-transcriptional levels (Dey et al., 2014). Additionally, it has been proved that lncRNAs are involved in physiological and pathobiological processes (Slaby et al., 2017). Emerging evidence shows that the expression profile of lncRNAs can serve as a diagnostic and prognostic tool for various tumors (Wang et al., 2017).

Human lncRNA OIP5-AS1 transcribes in antisense (AS) OIP5. It has been reported that OIP5-AS1 inhibits the proliferation of various malignant tumors (Dai et al., 2018). Moreover, lncRNA OIP5-AS1 can also play a carcinogenic role in several cancers. For instance, lncRNA OIP5-AS1 improves the tumorigenesis of hepatoblastoma via silencing of ZEB1 (Zhang et al., 2018). OIP5-AS1 promotes the progression of cervical cancer and acts as a negative prognostic factor for gastric cancer (Yang et al., 2019). It has been also indicated that OIP5-AS1 up-regulation is a carcinogenic factor in colorectal cancer (Zou et al., 2018). Despite the important function of OIP5-AS1 in tumors, its role in hemangioma remains not well-known. This study aimed to investigate effects of abnormal expression of OIP5-AS1 on the biological behavior of endothelial cells in hemangioma.

## Materials and Methods

### Tissue Samples

Tissue samples were collected from normal skin tissues (*n* = 31) from Affiliated Hospital of Armed Police Logistics College, and tissues from patients, who have been diagnosed as hemangioma at regressive stage (*n* = 40) and proliferative stage (*n* = 56). Prior were included in this study. All patients have signed the informed consent. The study has been approved by the Ethics Committee of Affiliated Hospital of Armed Police Logistics College.

### Cell Culture and Transfection

Hemangioma endothelial cells (HemECs) were purchased from Shanghai Oto Biotechnology Co., Ltd. (Shanghai, China), and preserved in an endothelial basal medium-2 (EBM-2; Cambrex Biotechnology, Vauxville, MD, United States) containing 10% FBS (Invitrogen, Shanghai, China). Specific shRNAs and NC-shRNA (sh-NC) were purchased from Santa Cruz Biotechnology Company (Dallas, TX, United States), which were applied to knock down OIP5-AS1 in HemECs, and pcDNA 3.1 vector (Guangzhou RiboBio Co., Ltd., Guangdong Province, China) was used to overexpress OIP5-AS1 or NOB1. Besides, miR-195-5p mimics and inhibitors were synthesized by RiboBio Co., Ltd., which were applied to overexpress or silence miR-195-5p.

### RNA Extraction and qRT-PCR

Firstly, the TRIzol reagent (Invitrogen, Grand Island, New York) was used to isolate total RNA. Transcriptase superscript III (Invitrogen, Grand Island, New York) was applied to complete reverse transcription. qRT-PCR was completed with Bio-Rad CFX96 system, and gene expression levels were detected by SYBR green. qRT-PCR primer sequences were as follows: OIP5-AS1, F: 5′-GGTCGTGAAACACCGTCG-3′, R: 5′-GTGGGGCATCCAGGGT-3′; NOB1, F: 5′-TGAAGGTCGACGTCACCG-3′, R: 5′-GGGGTGCCAGGTGATC-3′; GAPDH, F: 5′-CAGTCACTACTCAGCTGCCA-3′, R: 5′-GAGGGTGCTCC GGTAG-3′. The condition of PCRs was as follows: 50°C for 2 min, 95°C for 8.5 min, and then followed with 95°C for 15 s, 60°C for 1 min, which were performed for 35 cycles. The extension was carried out at 95°C for 1 min, 55°C for 1 min, and 55°C for 10 s. GAPDH serves as an internal reference to analyze the gene expression levels. The experiments were performed in triplicate for each condition.

### Cell Proliferation Assay

The cells were seeded into 24-well plates (3000/well) and cultured for about 48 h. Then, the medium was replaced by MTT reagent and the blue crystal was dissolved with dimethyl sulfoxide. Finally, cell activity was detected with absorbance at 570 nm. For colony formation assay, cells were seeded into 6-well plates (3000/well) and cultured in DMEM containing 10% FBS at 37°C. 2 weeks later, cells were washed with PBS and fixed with methanol for 0.5 h. Fixed clones were stained with 1% crystal violet and the number of clones was calculated manually. All experiments were performed in triplicate.

### Cell Migration and Invasion Test

Firstly, the treated cells were seeded into 6-well plates and cultured for 3 days. Matrigel (1:20, BD Corning) was added to Transwell plate for around 2 h before cell inoculation. Next, serum-free medium was applied to harvest cells and 1 × 10^5^/mL cells were added to the upper compartment. Then, 750 μl medium containing 10% FBS was added into the lower compartment. After incubation at 37°C with 5% CO^2^ for 12 h, the invaded cells were fixed with methanol and stained with 0.1% crystal violet in the darkroom. Trials for all samples were duplicated for three times. Cell migration ability was analyzed via Transwell’s test, which was carried out in accordance with methods reported in previous studies. Trials for all samples were duplicated for three times.

### Flow Cytometry Analysis

Cells were transfected with sh-OIP5-AS1 and sh-NC, respectively, and harvested post 2 days. The apoptotic rate was analyzed via flow cytometry analysis with Annexin V: FITC Apoptotic Detection Kit (BD Bioscience, United States) in accordance with the manufacturer’s instructions. Trials for all samples were duplicated for three times.

### Endothelial Tubules Formation Assay

Hemangioma endothelial cells were seeded into 96-well plate and incubated for 6 h at 37°C with 5% CO^2^ before transfection. Matrigel (BD Biosciences, San Jose, CA, United States) and ECM were mixed in a ratio of 1:2. 50 μl mixture was added into the holes of 96-well plate. Matrigel was coagulated via incubation at 37°C for 20 min. The tubules formation was photographed under a microscope and quantified with Image J software. Trials for all samples were duplicated for three times.

### RNA FISH

RNA FISH kit was purchased from Guangzhou RiboBio Co., Ltd. (Guangzhou, China). RNA FISH probes mixtures of OIP5-AS1, 18S or U6 were synthesized and produced by Guangzhou RiboBio Co., Ltd. RNA FISH was carried out as described previously. The nucleus was reversely stained with 18 DAPI (RiboBio). High resolution images were obtained with Laser scanning confocal microscopy (Zeiss, Jenna, Germany).

### Subcellular Components Detection

PARIS^TM^ kit (Ambion, Austin, TX, United States) was applied to analyze nucleus components. Cell suspension was prepared in cell component isolation buffer after 1 × 10^7^ cells were collected. The cells were incubated on ice for 10 min prior to the next trial. The upper solution was discarded after centrifugation, nucleus particles were preserved with the cell division buffer for RNA extraction. Trials for all samples were duplicated for three times.

### RIP Detection

RNA immunoprecipitation was carried out with the EZ-Magna RIP kit (Millipore, Billerica, MA, United States) according to the guidelines. HemECs were scraped off from the culture plate and lysed in 100% RIP Lysis Buffer. Cell extracts were stored in RIP buffer containing magnetic beads which could adsorb human anti-Ago2 antibody (1:1200). Normal mice IgG (Millipore) was used as the NC group. Additionally, RNA concentration was measured with NanoDrop Spectrophotometer (Thermo Fisher Scientific), and RNA quality was analyzed with Bioanalyzer (Agilent, Santa Clara, CA, United States). All experimental samples were made in triplicate.

### Pull-Down Test

MiR-195-5p, miR-195-5p-Mut, and NC were biotinylated into Bio-miR-195-5p-WT, Bio-miR-195-5p-Mut, and Bio-NC by Shanghai Jima Pharmaceutical Technology Co., Ltd. (Shanghai, China), which were transfected into HemECs, respectively, and the cells were collected and lysed post 48 h. The cells were incubated in Dynabeads M-280 Streptavidin (Invitrogen, CA, United States) for 10 min, and then washed with buffer. Quantitative analysis of binding RNA was carried out with qRT-PCR.

### Western Blot Analysis

Firstly, proteins were separated by SDS–PAGE and transferred to PVDF membranes (Millipore, Billericay, MA, United States). Then the membrane was blocked with bovine serum albumin (Sigma-Aldrich, St. Louis, MO, United States). Next, the membrane was incubated with primary antibodies at 4°C overnight, which was followed by the secondary antibody incubation at room temperature for 1 h. The main antibodies included cleaved caspase 3 (Abcam, ab2302, dilution ratio, 1:2000), cleaved caspase 9 (Abcam, ab2324, dilution ratio, 1:2000), anti-NOB1 (Abcam, ab224619, dilution ratio, 1:2000) and anti-GAPDH (Abcam, ab181602, dilution ratio, 1:3000). Secondary antibodies were rabbit-anti-mouse IgG H&L (abcam, ab6728, dilution ratio, 1:2000) or Goat Anti-Rabbit IgG H&L (abcam, ab6721, dilution ratio, 1:2000). ECL Chemiluminescence Detection System (Thermo Fisher Scientific, Rochester, New York) was applied to visualize protein bands. Finally, the bands were exposed with X-ray film. The experiments were performed for three times.

### Statistical Analysis

SPSS 19.0 software (SPSS Inc., Chicago, IL, United States) was used for statistical analysis. The data are shown with mean value ± standard deviation, which were outcomes of more than two experiments. Student’s t test (two-tailed, equal variance) was applied to analyze the differences between the two groups, and one-way ANOVA was used to perform multi-factor comparison. The correlation of OIP5-AS1, miR-195-5p, and NOB1 was detected with Spearman related analysis. *P* < 0.05 was deemed as statistically significant.

## Results

### The Effects of Silencing OIP5-AS1 on the Proliferation and Apoptosis of HemECs

**Figure 1:**
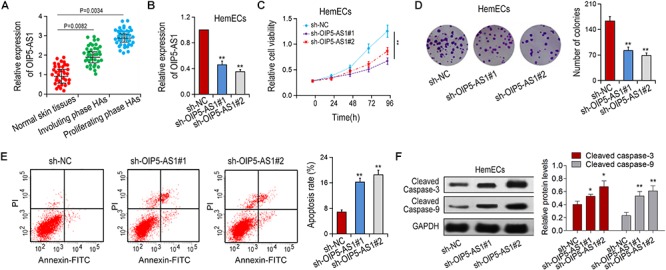
Effects of OIP5-AS1 on proliferation and apoptosis of HemECs cells. **(A)** The expression of OIP5-AS1 in different tissues was detected by qRT-PCR. Normal skin tissues, *n* = 34; Involuting phase HAs, *n* = 36; proliferating phase HAs. **(B)** sh-OIP5-AS1 and sh-NC were transfected into HemECs the expression of OIP5-AS1 was determined by qRT-PCR. **(C,D)** Cell proliferation of HemECs transfected with sh-NC or sh-OIP5-AS1 was examined by MTT assay and colony formation assay. **(E)** Cell apoptosis was detected by flow cytometry analysis. **(F)** Apoptotic rates were detected in HemECs cell by Western blot analysis. ^∗^*P* < 0.05 and ^∗∗^*P* < 0.01 vs. controls.

To investigate the biological function of OIP5-AS1 in hemangioma, we first detected the expression of OIP5-AS1 in three different types of tissues (Figure 1A). It was shown that the expression of OIP5-AS1 in proliferative tissues was significantly upregulated compared with that in regressive tissues and normal tissues. Next, we determined the effect of OIP5-AS1 on the growth of HemECs. We knocked down the expression of OIP5-AS1 in HemECs via shRNA transfection (Figure 1B) and detected the effects of OIP5-AS1 on the proliferation of HemECs by MTT and colony formation assays. The results showed that knockdown of OIP5-AS1 significantly inhibited the cell activity of HemECs (Figure 1C). Similarly, silencing of OIP5-AS1 significantly reduced the colony forming efficiency of HemECs (Figure 1D). Moreover, knockdown of OIP5-AS1 enhanced apoptosis of HemECs (Figure 1E) as detected by flow cytometry. Consistently, down-regulation of OIP5-AS1 increased the expression of apoptotic proteins (cleaved caspase-3 and cleaved caspase-9) (Figure 1F). Taken together, these results suggest that silencing of OIP5-AS1 inhibits cell proliferation and induces cell apoptosis.

### The Effects of Silencing of OIP5-AS1 on Migration, Invasion, and Angiogenesis of HemECs

**Figure 2:**
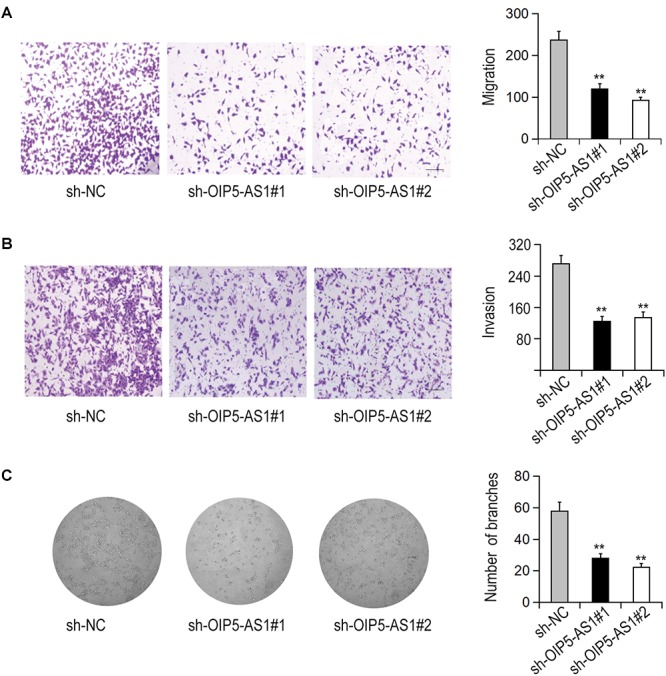
Effects of OIP5-AS1 on the migration, invasion, and vasoformation of HemECs. **(A,B)** The migration and invasion of HemECs were detected by Transwell assay after knockdown of OIP5-AS1 (Bar = 200 μm). **(C)** The tube formation of HemECs after OIP5-AS1 knockdown was detected by Tube formation assay.

The effects of OIP5-AS1 on the migration and invasion of HemECs were detected with Transwell test. The results showed that silencing of OIP5-AS1 significantly decreased the migration and invasion of HemECs (Figure 2A,B). We then investigated the effect of OIP5-AS1 on tubule formation of HemECs. It was shown in that the complicated branching tubule network structure of HemECs was turned to be simple after silencing of OIP5-AS1 (Figure 2C).

### OIP5-AS1 Binds to miR-195-5p in HemECs

**Figure 3:**
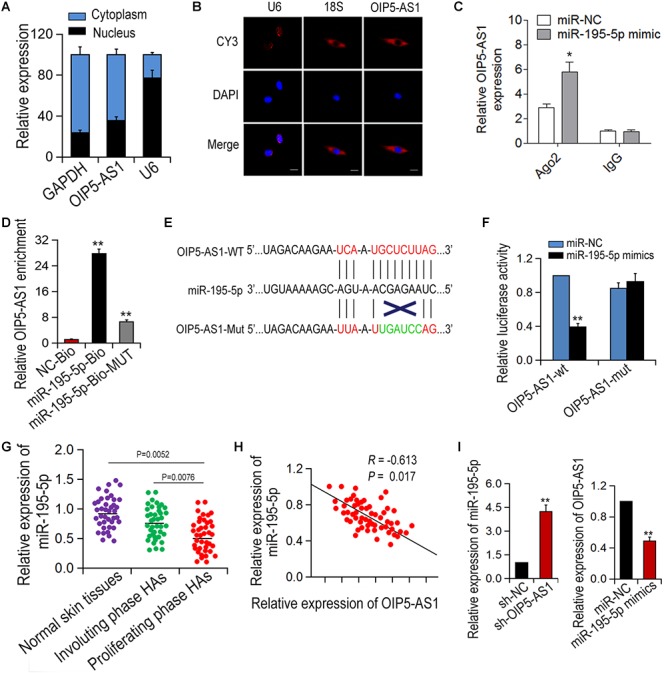
OIP5-AS1 can bind with miR-195-5p in HemECs. **(A,B)** The localization of OIP5-AS1 in HemECs was examined by Subcellular fractionation assay and RNA FISH. **(C,D)** RIP assay was conducted in HemECs to explore the combination between OIP5-AS1 and miR-195-5p. **(E)** The binding sites between OIP5-AS1 (WT and Mut) and miR-195-5p. **(F)** The interaction between OIP5-AS1 and miR-195-5p was performed by dual-luciferase assay. **(G)** The expression of miR-195-5p was detected in different tissues. **(H)** The correlation between OIP5-AS1 and miR-195-5p was examined by Spearman’s analysis (*n* = 56). **(I)** Expressions of OIP5-AS1 and miR-195-5p after transfection with OIP5-AS1 knockdown or miR-195-5p overexpression. ^∗^*P* < 0.05 and ^∗∗^*P* < 0.01 vs. controls.

Next, we investigated the molecular mechanism of the action of OIP5-AS1 in hemangioma. It has been reported that OIP5-AS1 can serve as ceRNA in human cervical cancer. We hypothesized that OIP5-AS1 may function in a similar way in HemECs. To verify this assumption, we first determined the locations of OIP5-AS1 via subcellular isolation analysis and RNA FISH assay and found that OIP5-AS1 was localized in the cytoplasm of HemECs (Figure 3A,B). Next, using Starbase database^1^ we predicted miRNAs that can bind to OIP5-AS1 and found miR-195-5p as a potential target. To verify the direct binding of OIP5-AS1 and miR-195-5p, RIP analysis was performed in HemECs containing Ago2 antibody. The result showed that compared with the control IgG, both OIP5-AS1 and miR-195-5p are with tendency to be ago2-rich beads (Figure 3C). It was also showed that OIP5-AS1 could be pulled down by bio-miR-195-5p-WT, but not by bio-miR-195-5p-MUT or Bio-NC via pull-down analysis (Figure 3D). Furthermore, luciferase reporter assay showed that miR-195-5p mimic could significantly reduce the luciferase activity of wild-type OIP5-AS1 while had no obvious effect of OIP5-AS1 mutant (Figure 3E,F). In addition, the miR-195-5p expression level in proliferative HA tissues was significantly lower compared with normal skin tissues and involuting phase HAs (Figure 3G). A negative correlation between OIP5-AS1 and miR-195-5p in proliferative HA tissues was revealed via Spearman correlation analysis (Figure 3H). The relationship of OIP5-AS1 and miR-195-5p in HemECs was further confirmed by qRT-PCR (Figure 3I). Taken together, these results suggest that OIP5-AS1 can bind to miR-195-5p.

### OIP5-AS1 Regulates NOB1 via Binding to miR-195-5p

**Figure 4:**
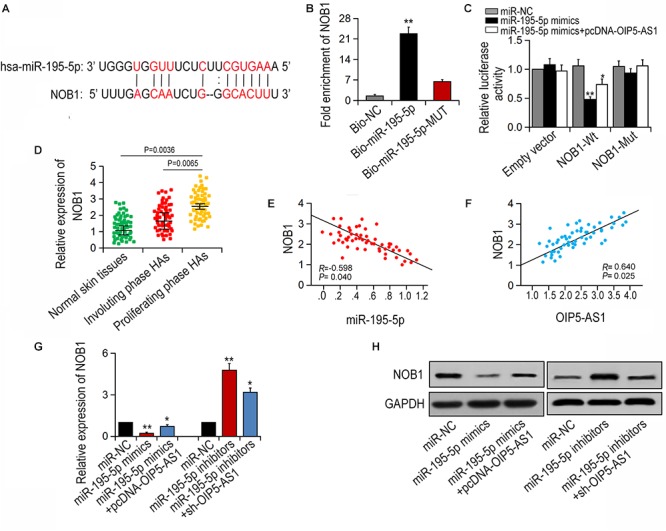
OIP5-AS1 regulates NOB1 through binding to miR-195-5p. **(A)** The binding sites between miR-195-5p and NOB1 were achieved by bioinformatics analysis. **(B)** The relation between miR-195-5p and NOB1 was conducted by pull-down assay. **(C)** The luciferase activity of NOB1 could be affected by miR-195-5p mimics. **(D)** The expression of NOB1 was detected in different tissues. **(E,F)** The correlations between miR-195-5p/NOB1 and OIP5-AS1/NOB1 were detected by Spearman’s analysis (*n* = 56). **(G,H)** Expression of NOB1 after miR-195-5p mimics and pcDNA-OIP5-AS1 as well as sh-OIP5-AS1 and miR-195-5p inhibitors were transfected into HemECs. ^∗^*P* < 0.05 and ^∗∗^*P* < 0.01 vs. controls.

NOB1 has been reported to be an important regulator in several cancers (Zhang et al., 2014; Yin et al., 2015; Wang et al., 2018). Using public database MiRBase^2^, NOB1 was initially predicted to be a potential target of miR-195-5p (Figure 4A). To determine the direct interaction between miR-195-5p and NOB1, pull-down assay was performed. It was shown that NOB1 could be pulled down by bio-miR-195-5p-WT, but not by bio-miR-195-5p-MUT or Bio-NC (Figure 4B). The interaction was further confirmed by double Luciferase reporter assay. It was shown that miR-195-5p mimics reduced the luciferase activity of wild-type NOB1, but did not have significant effect on the luciferase activity of NOB1 mutant. And the reduced luciferase activity could be restored by OIP5-AS1 overexpression (Figure 4C). In addition, NOB1 expression was the highest in HA tissues at the proliferative stage (Figure 4D). A negative correlation of NOB1 and miR-195-5p and a positive correlation of OIP5-AS1 and NOB1 were found (Figure 4E,F). Next, we examined the effects of OIP5-AS1 or miR-195-5p on NOB1 expression. qRT-PCR and western blot analysis showed that transfection with miR-195-5p mimic significantly reduced the mRNA and protein levels of NOB1, and the inhibition was reversed by overexpression of OIP5-AS1. In contrast, treatment with miR-195-5p inhibitors increased the mRNA and protein level of NOB1, and the enhancement was restrained following silencing of OIP5-AS1 (Figure 4G,H). Taken together, these data suggest that OIP5-AS1 regulates NOB1 expression by blocking miR-195-5p in HemECs.

### Effects of OIP5-AS1-miR-195-5p-NOB1 Pathway on the Cellular Activity of HemECs

**Figure 5:**
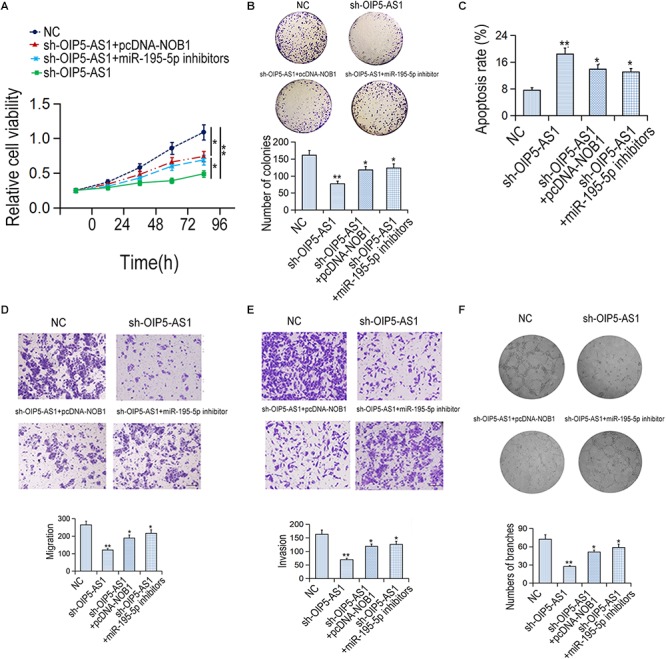
The effects of OIP5-AS1/miR-195-5p/NOB1 pathway on HemECs cell migration, invasion and apoptosis. **(A,B)** The proliferative rate of HemECs was detected by MTT and colony formation assays. **(C)** The apoptotic rate was examined by flow cytometry assay. **(D,E)** The migration and invasion of HemECs cells were detected by Transwell assay. Bar = 200 μm. **(F)** The vasoformation of HemECs cells was examined by tube formation assay. ^∗^*P* < 0.05 and ^∗∗^*P* < 0.01 vs. controls.

Rescue experiment was carried out to verify the effect of OIP5-AS1-miR-195-5p-NOB1 axis on the activity of HemECs. MTT assay and colony formation assay showed that the proliferation ability of HemECs was reduced after down-regulation of OIP5-AS1, which was reversed by transfection with pcDNA-NOB1 or miR-195-5p inhibitors (Figure 5A,B). Flow cytometry analysis showed that the increased apoptosis caused by sh-OIP5-AS1 was reduced by transfection with pcDNA-NOB1 and miR-195-5p inhibitors (Figure 5C). Transwell assays showed that the migration and invasion ability of HemECs decreased by OIP5-AS1 knockdown was restored by transfection with pcDNA-NOB1 or miR-195-5p inhibitors (Figure 5D,E). Tubular formation analysis revealed that the inhibitory effect of sh-OIP5-AS1 on angiogenesis was restored by pcDNA-NOB1 and miR-195-5p inhibitors (Figure 5F).

## Discussion

It was reported that OIP5-AS1 is involved in various biological activities, especially in cell proliferation, invasion, and migration processes (Zhang et al., 2018; Chen et al., 2019). Here, we attempted to investigate the effect of OIP5-AS1 on the cell activity of HemECs. The ceRNA pattern is a biological pathway of great significance, and the lncRNA mediated with miRNAs can be involved in all tumor processes via the regulation of ceRNA. Substantial evidence on the role of lncRNAs in the complex processes of human diseases has been provided in many studies. CeRNAs have been identified as a marker of diagnosis or prognosis in many reports. lncRNAs can participate in the ceRNA pattern and regulate gene expression in some cases. In this study, we hypothesized that OIP5-AS1 might be a ceRNA in HemECs.

miRNAs have been identified as independent regulators in various tumors, which also play a specific role on the ceRNA axis (Hata and Lieberman, 2015). Generally, miRNAs were considered as the target gene of lncRNAs in the regulation of tumorigenesis, which participate in the biological processes of many human tumors via interaction with lncRNAs or targeting mRNAs. MiR-129-5p is involved in ceRNA pathway in colon cancer (Wu et al., 2018). MiR-181a-5p is involved in the progression of multiple myeloma (Chen L. et al., 2018). The interaction of miRNA and mRNA in tumors was demonstrated by the above examples.

The regulatory model of ceRNA (OIP5-AS1-miR-195-5p-NOB1) was shown in our study and we performed our investigation. Firstly, we silenced OIP5-AS1 to detect the changes of cell activity (proliferation, migration, and invasion) in HemECs. It was shown that OIP5-AS1 silencing could significantly inhibit the proliferation, metastasis as well as angiogenesis of HemECs. We further discussed the potential molecular mechanism of OIP5-AS1 in HemECs. OIP5-AS1 was found in the cytoplasm of HemECs. RIP experiments and pull-down analysis were carried out to verify this hypothesis. It was confirmed that OIP5-AS1 could bind to miR-195-5p, and the binding sites of OIP5-AS1 and miR-195-5p was searched with bioinformatics analysis, which was also applied to determine the binding sites of miR-195-5p and NOB1. NOB1 is a zinc protein involved in ribosome biogenesis and proteolysis. Numbers of studies have shown that NOB1 plays an essential role in cancer development. It has been reported that downregulation of NOB1 inhibits cell proliferation in prostate cancer, oral squamous cell carcinoma, and osteosarcoma. In this study, double luciferase reporters were applied to clarify the composition relationship of these three genes. We found that OIP5-AS1 regulates NOB1 expression positively via blocking the expression of miR-195-5p. Finally, the effects of OIP5-AS1-miR-195-5p-NOB1 on the proliferation, apoptosis, migration and angiogenesis of HemECs were verified with rescue experiments.

## Conclusion

Taken together, this study reveals a new ceRNA pathway in HemECs. The upstream molecular mechanism of the OIP5-AS1-miR-195-5p-NOB1 axis would be further investigated. This study is of great value in the investigation of additional biological pathways of hemangioma endothelial cells.

## Data Availability

The raw data supporting the conclusions of this manuscript will be made available by the authors, without undue reservation, to any qualified researcher.

## Ethics Statement

All patients signed an informed-consent document for diagnosis and research on tissue specimens before being enrolled in the project. All subjects gave written informed consent in accordance with the Declaration of Helsinki principles. The protocol was approved by the Ethics Committee of the China-Japan Union Hospital of Jilin University.

## Author Contributions

YL performed the conception and intellectual the input. JZ designed and performed the experiments. TZ drafted the manuscript. LT performed the statistical analyses and interpreted the data. All authors read and approved the final manuscript.

## Conflict of Interest Statement

The authors declare that the research was conducted in the absence of any commercial or financial relationships that could be construed as a potential conflict of interest.

## Footnotes

1 http://www.lncrnablog.com/starbase-v2-0-for-decoding-rna-interaction-networks/

2 http://www.mirbase.org/
